# Chemical Camouflage Induced by Diet in a Pest Treehopper on Host Plants

**DOI:** 10.3390/plants13020216

**Published:** 2024-01-12

**Authors:** Luan Dias Lima, Amalia Victoria Ceballos-González, Amanda Prato, Adriano Cavalleri, José Roberto Trigo, Fábio Santos do Nascimento

**Affiliations:** 1 Faculdade de Filosofia, Ciências e Letras de Ribeirão Preto, Departamento de Biologia, Universidade de São Paulo—USP, Ribeirão Preto 14040-901, SP, Brazil; aceballos@usp.br (A.V.C.-G.); amandaprato@usp.br (A.P.); fsnascim@usp.br (F.S.d.N.); 2 Instituto de Ciências Biológicas, Universidade Federal do Rio Grande—FURG, Rio Grande 96203-900, RS, Brazil; cavalleri_adriano@yahoo.com.br; 3 Departamento de Biologia Animal, Instituto de Biologia, Universidade Estadual de Campinas—UNICAMP, Campinas 13083-970, SP, Brazil

**Keywords:** ant–plant–herbivore interactions, chemical similarity, chemical strategy, cuticular hydrocarbons, multitrophic interaction, mutualism

## Abstract

Ants patrol foliage and exert a strong selective pressure on herbivorous insects, being their primary predators. As ants are chemically oriented, some organisms that interact with them (myrmecophiles) use chemical strategies mediated by their cuticular hydrocarbons (CHCs) to deal with ants. Thus, a better understanding of the ecology and evolution of the mutualistic interactions between myrmecophiles and ants depends on the accurate recognition of these chemical strategies. Few studies have examined whether treehoppers may use an additional strategy called chemical camouflage to reduce ant aggression, and none considered highly polyphagous pest insects. We analyzed whether the chemical similarity of the CHC profiles of three host plants from three plant families (Fabaceae, Malvaceae, and Moraceae) and the facultative myrmecophilous honeydew-producing treehopper *Aetalion reticulatum* (Hemiptera: Aetalionidae), a pest of citrus plants, may play a role as a proximate mechanism serving as a protection against ant attacks on plants. We found a high similarity (>80%) between the CHCs of the treehoppers and two of their host plants. The treehoppers acquire CHCs through their diet, and the chemical similarity varies according to host plant. Chemical camouflage on host plants plays a role in the interaction of treehoppers with their ant mutualistic partners.

## 1. Introduction

Plants exhibit a range of defenses, including chemical and physical mechanisms, as well as adaptations to attract predators and parasites of herbivores [1,2]. Ants are regarded as the most effective defensive strategy in plant biotic defense for plants lacking effective antiherbivore chemical defenses [1]. Ants primarily defend plants by collecting extrafloral nectar and honeydew, secreted by both plants and insects as rewards for ants, while consequently preying on herbivorous insects [3,4,5]. Herbivorous insects suffer a strong selective pressure on vegetation, where ants stand out as one of their main predators [6,7,8]. The cuticles of social insects, as well as those of other insects and plants, contain chemical cues called cuticular hydrocarbons (CHCs). These compounds, composed solely of hydrogen and carbon atoms, serve the primary functions of preventing desiccation and providing protection, while they also carry information that can be perceived by insects [7,9,10]. The CHCs are complex mixtures of straight-chain alkanes and alkenes and methyl-branched hydrocarbons, and they are used by social insects for recognition and communication, influencing the life histories of insects [11,12,13]. There is evidence that CHCs may be acquired by insects through abiotic and biotic environmental factors, including diet [14,15,16,17]. This characteristic makes insects good models for studies of chemical ecology. Even though ants are dominant and act as predators, some organisms are able to use chemical strategies with their CHCs and thus are able to maintain specific interactions called myrmecophily. These organisms are called myrmecophiles, and at least part of their life history depends on interactions with ants [18,19,20]. These interactions occur since ants forage on substrates and can influence the life of herbivorous insects, with interactions including commensalism, mutualism, or parasitism depending on the cost for the ant colonies (e.g., [21,22,23]).

There are mutualistic interactions called trophobiosis where herbivorous myrmecophiles such as hemipterans and lepidopterans produce liquid rewards rich in sugar that attract and appease ants during interactions where ants offer protection and grooming in exchange [22,24,25,26,27,28,29,30]. However, ants may prey on honeydew-producing mutualistic partners (e.g., [30,31,32]). Thus, even though treehoppers may be recognized by learning, their CHCs may play a role in avoiding ant attacks. Few studies have examined the possibility that treehoppers with gregarious habits may use an additional strategy called chemical camouflage to reduce ant aggression (i.e., [4,33]). Chemical camouflage is a strategy that decreases the detectability and recognition of an emitter by a receiver because the chemical cues of the emitter blend with a background, causing no reaction in the receiver [4,33,34,35]. Even though many studies have shown that insect, including agricultural pests, avoid aggression by providing honeydew to ants [30], none of these studies have considered how highly polyphagous insect pests of plants could avoid ant aggression using chemical strategies. Therefore, the precise recognition of the strategies is essential for a better understanding of the ecology and evolution of the mutualistic interactions between myrmecophiles pests of plants and ants.

*Aetalion reticulatum* (Linnaeus, 1767) (Hemiptera: Aetalionidae) is a facultative myrmecophilous honeydew-producing treehopper with gregarious habits and a polyphagous diet, being a pest of citrus plants in South America [36,37]. Recently, ref. [29] studied membracids and aetalionids in mutualistic interactions with ants and showed that a sugary reward attracts and reduces the risk of predation by ants. These authors also suggested that the CHCs of treehoppers may play a role in providing chemical camouflage in these interactions as they can blend with the background and go unrecognized as prey. Previous studies have demonstrated that the chemistry of plants influences multitrophic interactions and the behavior of ants [38,39]. Moreover, there is evidence for the plasticity of CHCs in herbivorous insects, indicating changes based on host plants [14,15,17]. This plasticity can serve as a chemical camouflage strategy [40,41,42]. Thus, we aimed to investigate whether there is a chemical similarity of CHC profiles of *A. reticulatum* treehoppers and their host plants that may protect the treehoppers against ant attacks. Our hypothesis was that the cuticular compounds of treehoppers would be diet-induced and resemble those of their host plants.

## 2. Results

**Figure 1:**
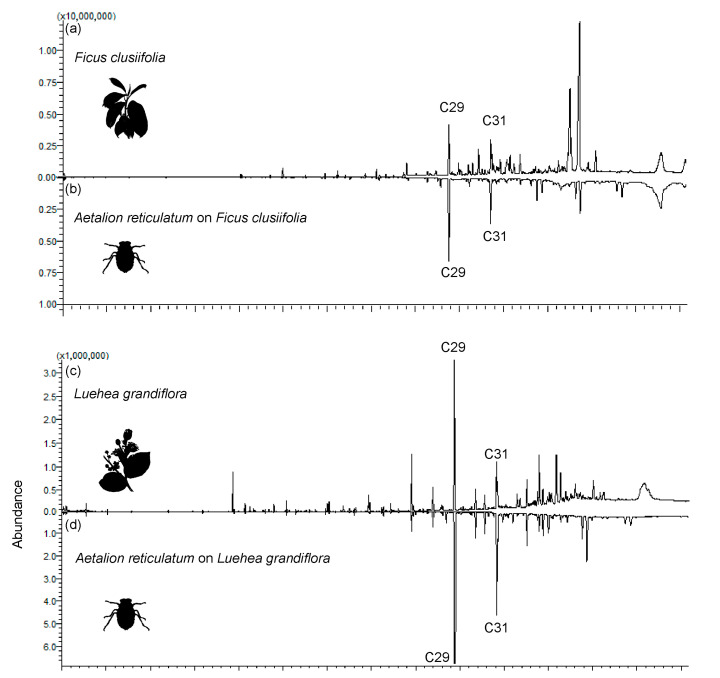

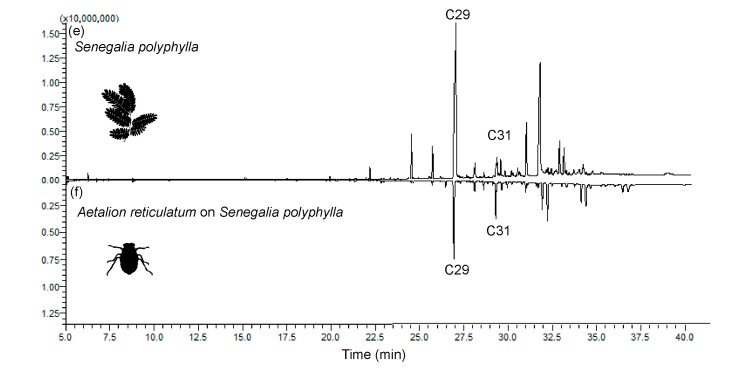
Chromatograms of cuticular compounds of the host plants *Ficus clusiifolia* (Moraceae), *Luehea grandiflora* (Malvaceae), and *Senegalia polyphylla* (Fabaceae) (**a**,**c**,**e**), and nymphs of *Aetalion reticulatum* that fed on these host plants (**b**,**d**,**f**). Compound identities can be found in Table 1.

**Figure 2:**
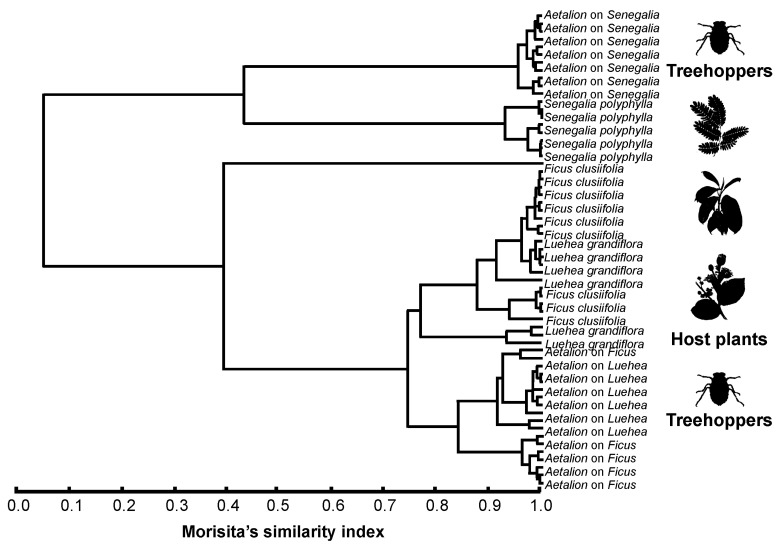
Hierarchical cluster analysis (Morisita’s similarity index) of the shared cuticular hydrocarbons of treehoppers and host plants.

The GC/MS analysis of cuticular compounds revealed a high degree of chemical similarity between *A. reticulatum* nymphs and their host plants (R_ANOSIM_ = 0.34; *p* < 0.001). In general, the treehoppers and their host plants had n-alkanes (C29 and C31) as their main components, and these compounds comprised between 33% and 78% of the relative abundance of the total compounds (Figure 1; Table 1). We found evidence for chemical camouflage (similarity > 80%) between *A. reticulatum* nymphs and their host plants *F. clusiifolia* (SI > 80%) and *L. grandiflora* (SI > 95%), but not between *A. reticulatum* nymphs and the host plant *S. polyphylla* (SI > 61%) (Figure 2).

The highest qualitative similarity was found between *A. reticulatum* nymphs and their host plant *L. grandiflora*, as the host plants showed 33 compounds and the nymphs feeding on this plant showed 37 compounds, with 19 being shared, representing 57.57% and 51.35% of the compounds on their respective cuticles (Figure 1; Table 1). Additionally, we found a significant positive correlation between the CHCs of *L. grandiflora* host plants and *A. reticulatum* nymphs (*r* = 0.9; *p* < 0.001). The *F. clusiifolia* host plants showed 16 compounds while the nymphs feeding on this plant showed 37 compounds, with 9 being shared, representing 56.25% and 24.32% of the compounds on their respective cuticles (Figure 1; Table 1). We also found a significant positive correlation between the CHCs of *F. clusiifolia* host plants and *A. reticulatum* nymphs (*r* = 0.7; *p* < 0.001). The *S. polyphylla* host plants showed 16 compounds, while the nymphs feeding on this plant showed 31 compounds, with 6 being shared, representing 37.5% and 19.35% of the compounds on their respective cuticles (Figure 1; Table 1). A significant positive correlation also emerged between the CHCs of *S. polyphylla* host plants and *A. reticulatum* nymphs (*r* = 0.4; *p* < 0.001). Moreover, the chemical profile of *A. reticulatum* feeding on the host plant *S. polyphylla* was more similar to the host plant than to the profiles of other treehoppers feeding on different host plants (Figure 2).

## 3. Discussion

We found a high degree of similarity (>80%) between the CHCs of *A. reticulatum* nymphs and some of their host plants in this study. Thus, our chemical analyses confirmed our initial hypothesis that the cuticular compounds of treehoppers would be diet-induced and resemble those of their host plants. It has been reported that host plants influence the CHC profile of herbivorous insects [11,14,15,17,43]. Moreover, certain insects acquire CHCs by feeding on host plants and blend with them as part of a chemical strategy called chemical camouflage, also known as chemical crypsis or phytomimesis [19]. Our results suggest that the studied treehoppers also employ chemical camouflage. This strategy was first suggested due to a possible similarity between the cuticular compounds of herbivorous insects and their host plants (see [34,35,43]), and it was later shown with chemical analyses and experiments in the caterpillar of *Biston robustum* Butler, 1879 (Geometridae) [40,41].

To our knowledge, this study is the first to show that chemical camouflage occurs in highly polyphagous pest insects. The treehopper studied belongs to the family Aetalionidae, and reports of this chemical strategy are still scarce for hemipterans. For example, the chemical camouflage strategy was also reported for the bug *Piezogaster reclusus* Brailovsky and Barrera, 2000 (Coreidae) [44,45] and treehoppers *Guayaquila xiphias* (Fabricius, 1803) [4] and *Tricentrus* sp. (Membracidae) [33]. Additionally, our results show that the proportion of n-alkanes, which are the main compounds on treehoppers, is similar to the host plants, although other compounds are not shared. The critical level of chemical similarity required for a chemical strategy to be effective remains unknown [46]. However, it is known that ants use plant hydrocarbons constituted mainly of n-alkanes to locate, identify, and protect plants [39,47]. Moreover, ants use n-alkanes to distinguish between myrmecophilous and nonmyrmecophilous partners to classify them into trophobionts and potential prey [48], and branched alkanes are used for recognition and may elicit aggression among non-nestmates [7]. Therefore, the treehoppers could be camouflaged in all the host plants studied here. Indeed, we did not conduct behavioral assays to confirm this in the field. However, the chemical similarities between treehoppers and the two host plants found in this study are higher than those already published, which have been demonstrated to function as a chemical strategy for treehoppers (i.e., [4]).

Moreover, it was recently reported that the myrmecophilous treehoppers *Enchenopa concolor* (Fairmaire, 1846) and *Enchenopa gracilis* (Germar, 1821) (Membracidae) have a chemical similarity with plants, and the latter also with the host plant *L. grandiflora* [49]. This similarity may trick butterflies to lay eggs directly on treehoppers instead of host plants [49], and it could also be used as a chemical camouflage strategy against ants. Chemical camouflage in treehoppers is diet-induced because they feed on their host plants and acquire their CHCs, resulting in a similar chemical profile that varies according to the host plant. Given that host plants influence the CHC profiles of herbivorous insects, which can be used as a chemical camouflage strategy [17,40,41,42], this is remarkable, considering that all ant-tended hemipterans are herbivorous [30]. The diet-induced chemical camouflage also occurs in other insects to avoid attacks from chemically oriented predators such as the larvae of the coleopteran *Chelymorpha reimoseri* Spaeth, 1928 (Chrysomelidae), lepidopteran caterpillars of *Mechanitis polymnia* (Linnaeus, 1758) (Nymphalidae), and four trophobiont species: *Allosmaitia strophius* (Godart, 1824), *Parrhasius polibetes* (Stoll, 1781), *Rekoa marius* (Lucas, 1857), and *Rekoa stagira* (Hewitson, 1867) (Lycaenidae) [42,46,50]. This kind of chemical camouflage may be widespread among herbivorous mutualistic insects due to its low cost [50], and the degree of chemical camouflage may change according to the plants they feed on (e.g., [4,40,41,42]). However, some insects may also acquire CHCs from plants at more than a trophic level [51], or they may biosynthesize their cuticular compounds and obtain a chemical similarity with their host plants [52]. Insects may also use physical contact to acquire compounds [16], but it has been reported that caterpillars that used chemical camouflage only acquired compounds through diet and not through physical contact [40].

Given that sugar rewards are used by trophobionts to attract predaceous ants that work as their bodyguards (e.g., [24,27,28,29,30]), our study showed the importance of chemical camouflage as an additional defensive mechanism for the herbivore mutualistic partners of ants to not become prey of their aggressive ant partners on plants. It has been shown that this strategy reinforces the mutualism of insects with ants on plants [4,33,42]. Moreover, as the trophobiont is chemically camouflaged, it may be perceived by ants as extrafloral nectaries (see [4]). Ants patrol plants with extrafloral nectaries and offer protection against herbivory [53], but most of the plants studied here do not possess extrafloral nectaries. In this context, ants would protect the herbivore that is chemically camouflaged, and this would influence in herbivory and consequently have a cost for plants. As the herbivore studied here is a pest of citrus plants [36,37], understanding chemical camouflage may be also important for integrated pest management strategies. Chemical camouflage may have allowed mutualistic insects to coexist with ants on plants despite the costs in the mutualistic interactions, and the cuticular composition of mutualistic partners plays a key role in decreasing attacks and increasing protection—and thus survival—in these insects [33,42].

## 4. Materials and Methods

### 4.1. Study Site and Organisms

We conducted collections during the dry season in the Ribeirão Preto campus of the University of São Paulo (21°16′37″ S, 47°85′92″ W), state of São Paulo, Brazil. Two seasons —a cold and dry season (April to September) and a warm and rainy season (October to March)—define the region’s climate [54]. The study site is in a transition between the Cerrado biome (Brazilian Savannah) and the Atlantic Forest and contains a reforestation site with several native and exotic trees and shrubs species [55].

We chose nymphs of the treehopper *Aetalion reticulatum* (Hemiptera: Aetalionidae) as these treehoppers usually engage in mutualistic interactions with ants on plants [29,36]. We opportunistically collected fourth-instar nymphs of the myrmecophilous treehopper with clean forceps and branches of three of their host plants: *Ficus clusiifolia* Schott (Moraceae), *Luehea grandiflora* Mart. and Zucc (Malvaceae), and *Senegalia polyphylla* (DC). Britton and Rose (Fabaceae) were used for chemical analyses. These host plants are frequently patrolled by dominant ant species on vegetation. This happens either because the host plants provide food or shelter for ants in exchange for protection in symbiotic relationships or because they show aggregations of honeydew-producing hemipterans which the ants interact with (see [56,57,58]).

### 4.2. Chemical Analyses

To determine whether polyphagous treehoppers acquired the CHCs through their diet, we compared the CHC profiles of nymphs and their host plants. We collected one *A. reticulatum* treehopper nymph from various aggregations and different branches of *F. clusiifolia* (n = 8 nymphs; n = 14 branches; not all branches contained treehoppers in this comparison), *L. grandiflora* (n = 10 nymphs; n = 10 branches), and *S. polyphylla* (n = 11 nymphs; n = 8 branches), which were also collected for analysis. After collection, the treehoppers samples were immediately frozen, and they were stored at a temperature of around −20 °C in a freezer until their cuticular components were extracted for chemical analyses the following day. The host plants samples were immediately brought to the laboratory for extractions. We assessed and identified the CHCs profiles of the studied treehopper nymphs and host plants via gas chromatography–mass spectrometry analysis (GC/MS), following [42]. We extracted the CHCs from individual treehopper nymphs and individual leaves of its host plant by immersing the samples for 1 min in 100 µL and 3 mL of hexane solvent, respectively (95% n-hexane, Macron Fine Chemicals, Radnor, PA, USA). To let the solvent evaporate, the extracts were stored in a flow chamber for 24 h. After that, the samples were resuspended in 40 µL of hexane, and 2 µL of this extract was injected (Splitless mode) in a gas chromatograph coupled with a mass spectrometer (Shimadzu, model QP2010, Kyoto, Japan), using a Rxi-1 column (Rxi-1 MS, 30 m × 0.25 mm × 0.25 μm, Restek Co., Bellefonte, PA, USA), with helium gas flow set at 1 mL/min. Initially set at 40 °C, the oven’s temperature increased by 15 °C/min until it reached 120 °C. The temperature then increased by 10 °C/min to 200 °C, 7 °C/min to 250 °C, and 5 °C/min to 320 °C for 6 min. The injector temperature was 250 °C. The detectors and the transfer lines had temperatures of 280 °C and 300 °C, respectively. We used the mass spectrometric fragmentation patterns (ion and molecular mass) [59] of the compounds, an alkane standard solution (C8-C20 and C21-C40, Fluka Analytical, Buchs, Switzerland), and the National Institute of Standards and Technology (NIST) mass spectra search program (version 2.2) Library database to identify the compounds.

### 4.3. Statistical Analyses

Compounds that were not present in more than half of the individuals belonging to a group, as well as compounds that contributed less than 0.5% to the total relative abundance, were excluded from the statistical analysis. We calculated the relative abundance of the compounds present in the cuticular extracts by considering the compounds as 100%. The quantity of each separated compound, expressed as a percentage of the total occurrence of the substance class, was then calculated using these data to determine relative abundances. We performed a cluster analysis based on Morisita’s similarity index (SI) to compare the CHC profiles of the studied species, following [42]. This index varies from 0% (no similarity) to 100% (complete similarity) [60]. We tested the significance of differences based on the percentage of similarity of CHC profiles between species using analysis of similarity (ANOSIM). R values were calculated between the groups in these analyses, ranging roughly from 0 (total similarity) to 1 (total difference) (see [61]). To explore potential correlations between the CHCs of host plants and treehoppers, we conducted correlation analyses using Pearson’s method. This involved calculating the mean relative abundance of CHCs for each host plant and correlating it with the mean relative abundance of CHCs of the treehoppers that fed on these host plants. All analyses were performed with PAST software (Version 4.13) [62]. We considered values of similarity above 80% between treehoppers and host plants as putative cases of the chemical camouflage strategy, as bioassays carried out by [4] showed this value to be sufficient to significantly reduce the detection of chemically camouflaged treehoppers against ants.

## 5. Conclusions

We show that treehoppers acquire CHCs from host plants and that this can serve as chemical camouflage for a treehopper which engages in mutualistic interactions with ants and acts as a pest for plants, suggesting a key role of this diet-induced chemical strategy in multitrophic interactions. Future research could conduct behavioral assays to experimentally confirm the results found here. Studies considering this chemical strategy are still scarce and could illuminate how the evolutionary process shaped mutualist interactions mediated by chemical strategies in a multitrophic context.

## Figures and Tables

**Table 1:** Cuticular compounds found on nymphs of *Aetalion reticulatum* and their host plants *Ficus clusiifolia* (Moraceae), *Luehea grandiflora* (Malvaceae), and *Senegalia polyphylla* (Fabaceae) (mean relative abundance ± standard deviation (*SD*)). (-) = compound not detected.

		Species					
Compounds	Retention Time	*Ficus clusiifolia*	*Aetalion reticulatum* on *Ficus clusiifolia*	*Luehea grandiflora*	*Aetalion reticulatum* on *Luehea grandiflora*	*Senegalia polyphylla*	*Aetalion reticulatum* on *Senegalia polyphylla*
n-C18	14.261	0.48 ± 0.23	-	0.10 ± 0.05	0.01 ± 0.01	-	-
n-C19	15.376	0.27 ± 0.13	-	0.10 ± 0.05	0.02 ± 0.01	-	-
Unknown 1	16.375	-	-	0.21 ± 0.09	0.02 ± 0.01	-	-
n-C20	16.509	0.55 ± 0.24	0.06 ± 0.02	0.21 ± 0.10	0.05 ± 0.02	-	-
n-C21	17.855	-	-	0.18 ± 0.11	0.03 ± 0.01	-	-
3MeC21	18.715	-	-	0.16 ± 0.06	0.02 ± 0.01	-	-
n-C22	18.785	0.49 ± 0.21	-	0.22 ± 0.11	0.05 ± 0.03	-	-
z-C23	20.008	-	-	0.43 ± 0.49	-	-	-
n-C23	20.106	-	-	1.51 ± 1.04	0.18 ± 0.07	0.59 ± 0.24	-
n-C24	21.028	0.43 ± 0.34	-	0.25 ± 0.13	0.06 ± 0.03	0.09 ± 0.03	-
C25:1	22.114	-	-	0.54 ± 1.23	-	-	-
n-C25	22.165	1.09 ± 0.74	0.07 ± 0.03	1.30 ± 1.01	0.24 ± 0.22	1.77 ± 0.39	0.18 ± 0.10
n-C26	23.329	0.53 ± 0.31	-	0.77 ± 0.65	0.12 ± 0.06	0.15 ± 0.03	-
C27:1	24.490	-	-	1.41 ± 2.80	-	-	-
n-C27	24.516	5.38 ± 2.81	0.48 ± 0.28	12.45 ± 4.63	2.62 ± 1.37	5.46 ± 0.43	0.68 ± 0.28
13,11MeC27	24.923	-	-	0.10 ± 0.05	-	0.16 ± 0.08	-
3MeC27	25.418	-	-	0.15 ± 0.08	-	0.11 ± 0.05	-
n-C28	25.705	2.20 ± 0.93	1.20 ± 0.24	3.18 ± 1.74	1.26 ± 0.27	3.56 ± 0.65	1.11 ± 0.24
Unknown 2	26.481	-	0.78 ± 0.56	-	1.52 ± 0.78	-	0.99 ± 0.32
z-C29	26.215	-	-	0.76 ± 1.20	-	0.14 ± 0.03	-
n-C29	26.938	41.50 ± 15.32	23.00 ± 5.10	43.89 ± 9.46	39.27 ± 5.43	36.10 ± 7.71	23.97 ± 2.16
15-;13-;11-;9MeC29	27.308	-	0.21 ± 0.09	-	0.38 ± 0.17	-	0.66 ± 0.47
7,11diMeC29	27.762	-	-	-	-	0.25 ± 0.19	-
3MeC29	27.821	-	-	-	0.22 ± 0.44	0.08 ± 0.03	-
n-C30	28.096	5.12 ± 1.86	2.73 ± 0.45	2.83 ± 1.44	3.14 ± 1.90	2.39 ± 0.25	2.57 ± 0.38
Unknown 3	28.860	-	0.86 ± 0.07	0.12 ± 0.06	0.52 ± 0.16	-	0.50 ± 0.12
C31:1 a	29.039	0.70 ± 1.28	-	0.64 ± 0.99	-	-	-
C31:1 b	29.422	-	-	0.32 ± 0.16	-	-	-
n-C31	29.547	35.54 ± 10.67	10.12 ± 3.56	15.51 ± 6.82	12.00 ± 3.81	1.45 ± 0.36	11.86 ± 2.44
Unknown 4	29.607	-	-	2.94 ± 2.02	-	-	-
15-;13-;9MeC31	29.644	-	2.76 ± 0.87	-	2.08 ± 1.20	-	2.46 ± 0.94
7MeC31	29.780	-	0.31 ± 0.27	-	0.16 ± 0.15	-	-
11,15diMeC31	29.960	-	0.89 ± 0.26	-	0.79 ± 0.26	-	1.54 ± 1.05
5,17diMeC31	30.219	-	1.68 ± 0.69	-	1.62 ± 0.55	-	1.65 ± 0.94
n-C32	30.464	1.68 ± 1.17	0.33 ± 0.08	0.98 ± 1.25	0.24 ± 0.12	-	0.27 ± 0.11
16,15,14MeC32	30.795	-	0.79 ± 0.17	-	0.38 ± 0.19	-	0.52 ± 0.18
3,11diMeC32	31.109	-	0.71 ± 0.46	-	-	-	0.62 ± 0.45
3,10diMeC32	31.540	-	-	-	-	-	0.38 ± 0.49
z-C33	31.339	1.00 ± 1.01	-	-	-	9.03 ± 0.66	-
n-C33	31.602	3.07 ± 2.18	1.20 ± 0.40	1.28 ± 0.91	0.94 ± 0.64	-	1.22 ± 0.62
Unknown 5	31.863	-	-	6.76 ± 4.35	-	38.67 ± 8.54	-
17-;15-;13MeC33	31.935	-	5.98 ± 0.68	-	5.38 ± 2.63	-	5.87 ± 1.52
15,19diMeC33	32.240	-	7.42 ± 2.64	-	7.77 ± 5.11	-	9.00 ± 3.07
5,17diMeC33	32.488	-	0.63 ± 0.21	-	0.54 ± 0.17	-	1.12 ± 0.66
15,14MeC34	33.043	-	1.00 ± 0.15	-	0.38 ± 0.16	-	0.82 ± 0.19
14,XdiMeC34	33.285	-	-	-	0.87 ± 0.42	-	-
4,XdiMeC34	33.313	-	1.58 ± 0.23	-	-	-	1.73 ± 0.30
3,10diMeC34	33.766	-	0.48 ± 0.23	-	-	-	0.75 ± 0.28
Unknown 6	33.835	-	0.40 ± 0.14	-	-	-	-
n-C35	33.988	-	0.13 ± 0.04	0.19 ± 0.33	-	-	-
17-;15MeC35	34.136	-	6.19 ± 0.85	-	4.00 ± 0.60	-	5.78 ± 0.44
15,19diMeC35	34.404	-	10.10 ± 1.82	-	8.12 ± 2.91	-	11.23 ± 1.91
Unknown 7	34.561	-	-	0.14 ± 0.07	-	-	-
5,17diMeC35	34.663	-	1.42 ± 0.50	-	0.66 ± 0.34	-	2.27 ± 1.23
n-C36	35.217	-	-	0.27 ± 0.29	-	-	-
13MeC36	35.247	-	0.74 ± 0.18	-	-	-	0.54 ± 0.20
4,17diMeC36	35.515	-	1.18 ± 0.30	-	-	-	0.64 ± 0.17
4,10diMeC36	36.049	-	0.32 ± 0.11	-	-	-	-
n-C37	36.368	-	-	0.12 ± 0.16	-	-	-
17-,15-,13MeC37	36.487	-	4.47 ± 0.73	-	2.01 ± 0.45	-	3.79 ± 0.45
17,21diMeC37	36.785	-	7.20 ± 0.56	-	2.33 ± 0.69	-	4.71 ± 1.39
Unknown 8	37.086	-	0.71 ± 0.45	-	-	-	0.58 ± 0.29
17-,15MeC39	39.538	-	0.25 ± 0.08	-	-	-	-
Unknown 9	39.923	-	1.67 ± 0.56	-	-	-	-

## Data Availability

All data underlying our study was deposited in the Figshare Digital Repository under the https://doi.org/10.6084/m9.figshare.24288082.v1.
